# Effects of switching from twice-daily tacrolimus to once-daily extended-release meltdose tacrolimus on cellular immune response

**DOI:** 10.3389/frtra.2024.1405070

**Published:** 2024-09-25

**Authors:** Moritz Anft, Panagiota Zgoura, Sarah Skrzypczyk, Michael Dürr, Richard Viebahn, Timm H. Westhoff, Ulrik Stervbo, Nina Babel

**Affiliations:** ^1^ Center for Translational Medicine and Immune Diagnostics Laboratory, Medical Department I, Marien Hospital Herne, University Hospital of the Ruhr-University Bochum, Herne, Germany; ^2^ Clinic for Internal Medicine, St. Anna Hospital Herne, Herne, Germany; ^3^ Clinic for Surgery, Knappschaftskrankenhaus Bochum, Bochum, Germany; ^4^ Berlin Institute of Health, Berlin-Brandenburg Center for Regenerative Therapies, and Institute of Medical Immunology, Charité—Universitätsmedizin Berlin, Corporate Member of Freie Universität Berlin, Humboldt-Universität zu Berlin Augustenburger Platz, Berlin, Germany

**Keywords:** immunity, Immunosuppression, kidney transplantation, tacrolimus, T cells

## Abstract

**Background:**

LCP-Tacro [LCPT], a novel once-daily, extended-release formulation of tacrolimus, has a reduced C_max_ with comparable AUC exposure, requiring a ∼30% dose reduction in contrast to immediate-release tacrolimus (IR-Tac). Once-daily LCPT in *de novo* kidney transplantation has a comparable efficacy and safety profile to that of IR-Tac with advantages in bioavailability and absorption. The present investigation intends to analyze the effects of conversion from IR-Tac to LCPT on phenotype and function of T-cells and B-cells.

**Methods:**

16 kidney transplant patients treated by triple standard immunosuppression with a stable graft function undergoing a switch from IR-Tac to LCPT were included in this observational prospective study. We measured the main immune cell types and performed an in-depth characterization of B cell, dendritic cells and T cells including regulatory T cells of the patients before, 4 and 8 weeks after IR-Tac to LCPT conversion using multi-parameter fl ow cytometry. Additionally, we analyzed T cells by assessing third-party antigens (Tetanus Diphtheria, TD)-reactive T cells, which could be analyzed by restimulation with tetanus vaccine.

**Results:**

Overall, we found no significant alterations following LCPT conversion for the most immune cell populations with a few cell populations showing transient quantitative increase. Thus, 4 weeks after conversion, more regulatory T cells could be measured in the patients with a significant shift from memory to naïve Tregs. Furthermore, we found a transient B cell expansion 4 weeks after conversion from IR-Tac to LCPT. There were no changes in the percentage of other basic immune cell types and the antigen-reactive T cells were also not altered after changing the medication to LCP-tacrolimus.

**Conclusion:**

Here, we demonstrate first insights into the immune system changes occurred under IR-Tac to LCPT conversion therapy in kidney transplant patients. While phenotypic and functional characteristics of the most immune cell populations did not change, we could observe an a transient expansion of regulatory T cells in peripheral blood following IR-Tac to LCTP conversion, which might additionally contribute to the overall immunosuppressive effect.

## Introduction

The triple immunosuppressive therapy including steroids, mycophenolate mofetil (MMF), and calcineurin inhibitors (CNI), remains the standard immunosuppression aimed at preventing allograft rejection. Tacrolimus has established itself as the preferred CNI in this immunosuppressive therapy after kidney transplantation (1–3). It works by inhibiting the activation of T cells through the Calcineurin-NFAT axis (4). Tacrolimus attaches to FK506-binding protein 12 (FKBP12), forming a complex that subsequently blocks calcineurin, an enzyme crucial for T cell activation. Consequently, this leads to decreased production of inflammatory cytokines, predominantly interleukin-2 (IL2), and a reduction in T cell proliferation (5). Although B cells also express calcineurin und NFAT, tacrolimus has no significant clinical effect on B cells directly, but CD4T cell-dependent activation of B cells is suppressed by tacrolimus (6–8).

One challenge with current tacrolimus medications is their poor solubility in water, which results in suboptimal bioavailability. In addition, there are major inter- and intra-individual differences in the absorption of tacrolimus, due to interactions with different ingredients in food or other medications or genetic factors such as polymorphisms in cytochrome P450 (9–11). To counteract these problems, LCPT (Envarsus XR® was developed as a new formulation of tacrolimus with prolonged drug release through MeltDose technology, which improves the absorption of fat-soluble drugs (12). Due to the increased bioavailability, it is possible to reduce the total amount of tarolimus in Envarsus by up to 30%–36% compared to IR-Tac or ER-Tac drugs (Prograf® and Advagraf®) (13). Clinical studies have shown that LCPT was not inferior to IR-Tac in terms of safety and efficacy in *de novo* kidney transplant patients (14, 15) and after the conversion from IR-Tac to LCPT in kidney transplant patients (12, 16). Due to the improved bioavailability, the pharmacokinetic profile has also been shown to be more consistent with a prolonged Tmax to Cmax concentration in LCPT vs. IR-Tac or ER-Tac (13, 17).

Reducing the total amount of tacrolimus by switching from immediate-release (IR) tacrolimus to prolonged-release (LCP) tacrolimus seems not to compromise the efficacy and safety of LCP tacrolimus compared to standard tacrolimus therapy. However, there is a lack of studies investigating the impact of the conversion to LCP tacrolimus on the cellular immune system. This exploratory study delves into a detailed examination of the immune system in patients both before and after conversion to LCP tacrolimus, aiming to analyze the effects of the reduced total amount of tacrolimus.

## Results

### Study design and patient characteristics

In this propspective exploratory study, we performed analysis of cellular immunity in 16 kidney transplant patients undergoing tacrolimus conversionfrom IR-Tac to LCPT for medical reasons. The study was approved by the ethical committee of the Ruhr University Bochum (ethical approval number 16-5649) and all study participants provided written informed consent. There were 6 women and 10 men with a median age of 55.4 years, and median transplant age of 0.8 months. Immunosuppressive therapy consisted of tacrolimus, MMF/MMA, and glucocorticoids (Supplementary Table S1). In 15 of the 16 patients, the daily tacrolimus dose could be reduced after conversion from IR-Tac to LCPT to achieve the same tacrolimus AUC as with IR-Tac. The median reduction was 30.9% (Supplementary Figure S1). The daily dose of MMF/MMA and glucocorticoids did not change significantly after conversion. Nevertheless, the number of patients with MMF/MMA treatment decreased from 14 to 11. One patient received additional threatment with azathioprine after conversion to LCPT.

### Stable phenotype and developmental stage of conventional T cells following Tac conversion

**Figure 1:**
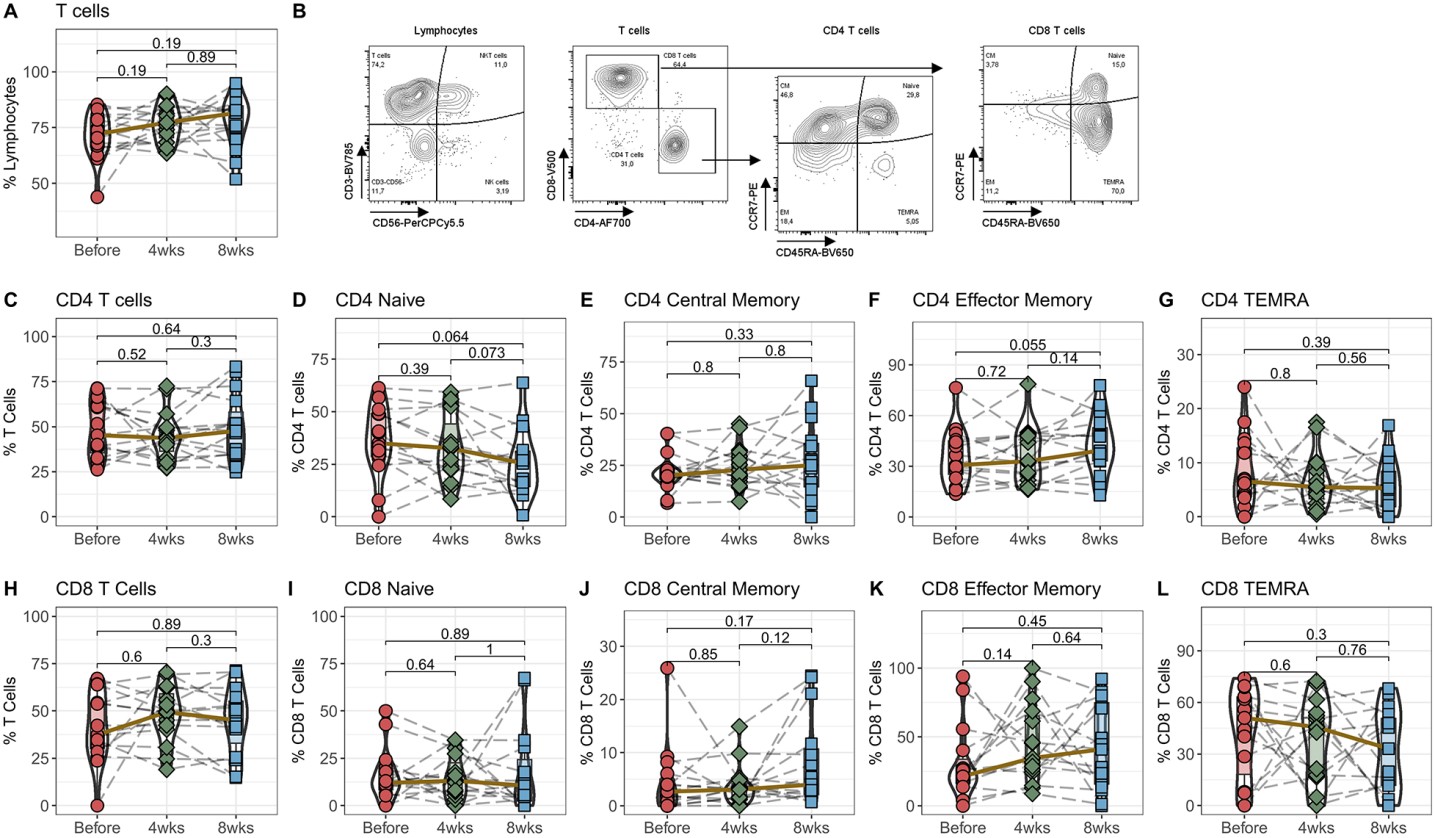
Effect of IR-Tac to LCPT conversion on T cells: Peripheral blood from 16 patients was drawn before IR-Tac to LCPT conversion, and after 4 and 8 weeks. Whole blood was stained with antibody panel for T cells and T cell memory subpopulations. Lymphocytes were identified by characteristic CD45/SSC-profile. T cells were identified by CD3^+^ expression **(A)** and further distinguished in CD4^+^ T helper cells **(C)**, CD8^+^ cytotoxic T cells **(H)**. CCR7 and CD45A were used to distinguished in naïve (**D+I**; CCR7^+^CD45RA^+^) and central memory (**E+J**; CCR7^−^CD45RA^−^), effector memory T cells (**F+K**; CCR7^+^CD45RA^+^), and T effector memory cells re-expressing CD45RA (**G+L**; TEMRA, CCD7^−^CD45RA^+^). Gating strategy see Panel **B** and Supplementary Figure S3. Mann-Whitney *U*-Test (paired), *p* < 0.05 = statistically significant.

In the first step, we quantified the percentage of T cells among all Lymphocytes in the peripheral blood. We did not see any significant differences in the general CD3-expressing T cells (Figure 1A) and the two subpopulations CD3^+^CD4^+^ T helper cells and CD3^+^CD8^+^ cytotoxic T cells (Figures 1C,H) 4 and 8 weeks after switching from IR-Tac to LCPT. To analyze the T cells in more detail, they were divided into naive T cells and the three memory subpopulations central memory, effector memory and TEMRA T cells based on their expression of CCR7 and CD45RA (Figure 1B). These T cell subpopulations also remain unchanged after switching the medication from standard IR-Tac to LCPT therapy with a reduced total dose of tacrolimus (Figures 1D–G, I–L).

### Initial expansion of regulatory T cells following Tac conversion

**Figure 2:**
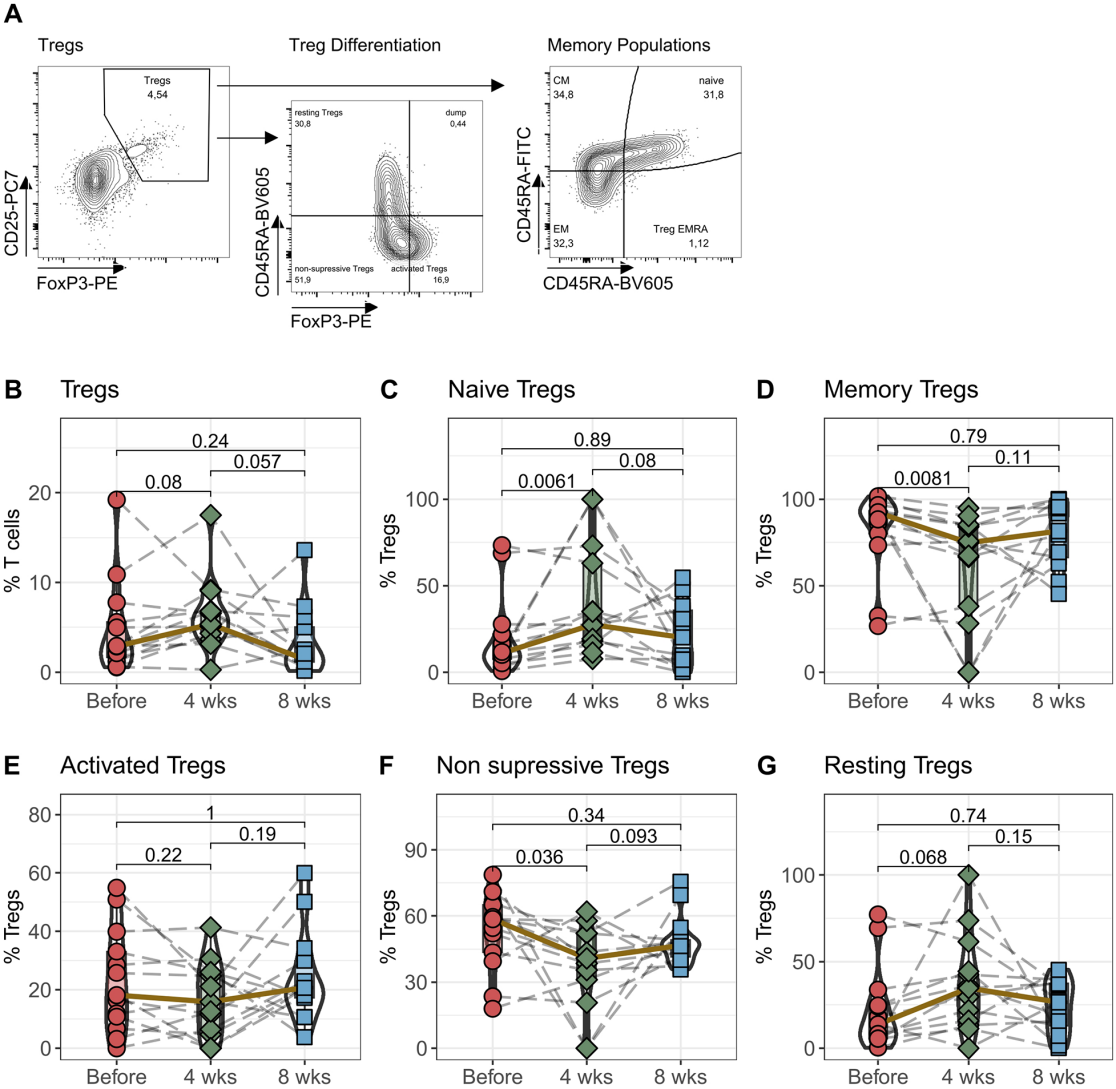
Regulatory T cells: Peripheral blood from 16 patients was drawn before IR-Tac ≥ LCPT conversion, and after 4 and 8 weeks. Isolated PBMC were stained with antibody panel for regulatory T cells. Regulatory T cells were identified as CD3^+^CD4^+^CD25^+^FoxP3+ T cells **(B)** and further distinguished by the expression of CCR7 and CD45A in naïve (**C**; CCR7^+^CD45RA^+^) and memory T cells (**D**; effector Memory (CCR7^+^CD45RA^+^), central memory (CCR7^−^CD45RA^−^) and Treg effector memory RA (Treg EMRA, CCD7^−^CD45RA^+^) cells combined). Furthermore, regulatory T cells were distinguished in activated (**E**; CD45RA^−^FoxP3^high^), non-suppressive (**F**; CD45RA^−^FoxP3^low^) and resting Tregs (**G**; CD45RA^+^FoxP3^low^). Gating strategy see Panel **A** and Supplementary Figure S4. Mann-Whitney *U*-Test (paired) *p* < 0.05 = statistically significant.

Regulatory T cells (Tregs) are doubly affected by medication with tacrolimus. They express NFAT and are therefore a direct target of calcineurin therapy. In addition, Tregs are particularly dependent on IL-2 for homeostasis, making them highly susceptible to changes in IL-2 plasma levels caused by tacrolimus. In the next step, we therefore analyzed the effects of switching from IR-Tac to LCPT therapy on Tregs, their activation- and memory-subpopulations (Figure 2A).

Following the switch to LCPT, the median percentage of CD3^+^CD4^+^CD25^+^FoxP3^+^ regulatory T cells among all CD3^+^ T cells increased 4 weeks later. However, by the eighth week, this proportion had reverted to roughly its original level before the switch (Figure 2B). Although noticeable, these changes were not statistically significant. Interestingly, upon examining the memory subpopulations, a notable pattern emerged: after 4 weeks, there was a significant rise in CCR7^+^CD45RA^+^ naïve Tregs alongside a significant decline in memory Tregs (comprising CM, EM, and TEMRA T cells) (Figures 2C,D). These changes normalized after eight weeks. Assessing the activation status of Tregs, no differences were found in the FoxP3^high^CD45RA^−^ activated Tregs. However, 4 weeks after switching to LCPT, there was a noteworthy decrease in non-suppressive Tregs (CD45RA^−^FoxP3^low^) and a borderline significant increase in resting Tregs (CD45RA^+^FoxP3^low^) (Figures 2E–G).

### Stable antigen-reactive T cell response following Tac conversion

**Figure 3:**
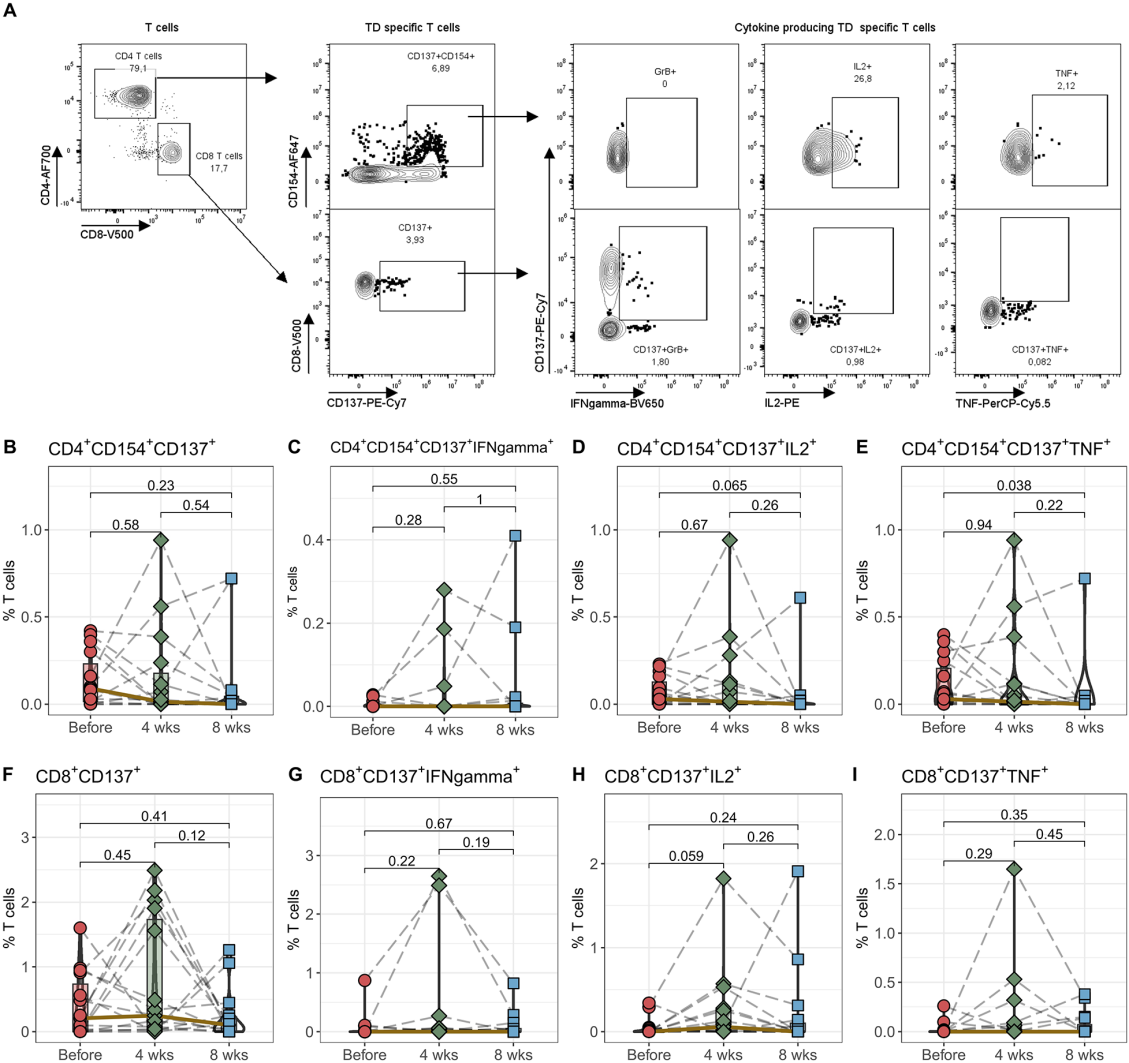
Antigen-reactiveT cells. Peripheral blood from 16 patients was drawn before IR-Tac ≥LCPT conversion, and after 4 and 8 weeks. Isolated PBMCs were stimulated for 16 h with TD vaccine+CD28. Antigen-reactive CD4^+^CD154^+^CD137^+^ T helper cells **(B–E)** and CD8^+^CD137^+^ cytotoxic T cells **(F–I)** were identified and further analyzed for the expression of granzyme B, IL-2 and TNF. Gating strategy see Panel **A** and Supplementary Figure S5. Mann-Whitney *U*-test (paired) *p* < 0.05 = statistically significant.

The immunosuppression following a transplant needs to strike a delicate balance. It must be potent enough to prevent rejection and at the same time keep the immune system sufficiently activated so that it can effectively recognize and eliminate pathogens. To find out how well the immune system is able to react after switching from IR-Tac to LCPT, the next step was to stimulate the cells with recall antigens (tetanus diphtheria, TD) and quantify their activation (Figure 3). We could not detect any differences in the percentage of TD antigen-reactive CD4^+^ T helper cells and CD8^+^ cytotoxic T cells by stimulation with TD after conversion to LCPT (Figures 3B,F). We measured the antigen-reactive immune response quality by examining the production of pro-inflammatory cytokines IFNγ, IL2, and TNF in activated T cells. There weren't significant shifts in cytokine production for CD4 T helper cells after 4 and 8 weeks (Figures 3C–E), nor in CD8 cytotoxic T cells (Figures 3G–I) following Tac conversion. TD-reactive TNF-producing CD4+ T cells showed a slight but not significant reduction after 8 weeks.

### Initial B cell expansion following Tac conversion

**Figure 4:**
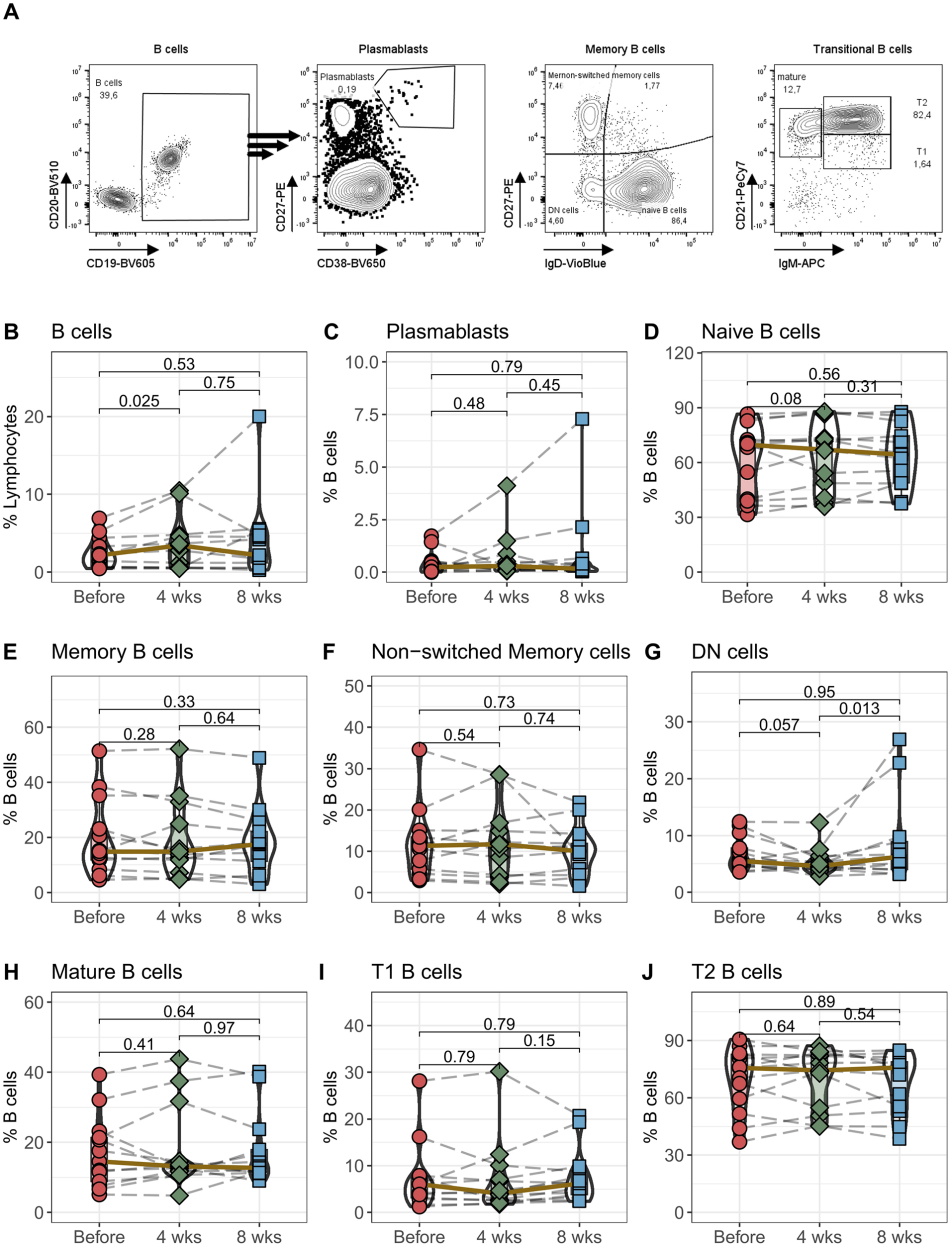
B cells: Peripheral blood from 16 patients was drawn before IR-Tac ≥ LCPT conversion, and after 4 and 8 weeks. B cells were identified as CD14^−^CD3^−^CD19^+^CD20^+^ B cells **(B)** and further characterized as plasmablasts (**C**; CD27^high^CD38^high^). B cells were further analyzed for memory subpopulations: Naïve B cells (**D**; IgD^+^CD27^−^), memory B cells (**E**; IgD^−^CD27^+^), non-switched memory (**F**; IgD^+^CD27^+^), and double negative B cells (**G**; DN, IgD^−^CD27^−^). B cell development stages were identified as mature B cells (**H**; IgM^−^CD21^+^), transitional 1 (**I**; T1, IgM^+^CD21^−^) and transitional 2 (**J**; T2, IgM^+^CD21^+^) B cells. Gating strategy see Panel **A** and Supplementary Figure S6. Mann-Whitney *U*-Test (paired) *p* < 0.05 = statistically significant.

The proliferation and differentiation of B cells is largely dependent on activation by CD4^+^ T helper cells. Although we did not detect any changes in CD4^+^ T cells, we examined B cells and their subpopulations in detail to determine whether there were changes in overall, naïve or memory B cells or in premature transitional B cells (Figure 4). Interestingly the percentage of B cells among all lymphocytes notably rose within the initial 4 weeks post-transition to LCPT, returning to standard levels by the 8-week mark (Figure 4B). However, there were no observable shifts in plasmablast numbers or in the subcategories of naïve, memory, or non-switched memory populations (Figures 4C–F). Notably, only the percentage of IgD and CD27 double-negative (DN) B cells showed a borderline significant decrease after 4 weeks (*p* = 0.057), followed by a significant increase after 8 weeks (Figure 4G). Finally, there were no discernible alterations in mature or in T1 and T2 B cell populations transition to LCPT (Figure 4H,I,J).

### No quantitative and qualitative alterations in the main immune cell subsets following Tac conversion

Considering the intricate interplay between different immune cell types, immunosuppressants can impact leukocyte subpopulations beyond their primary target, such as CI tacrolimus. Consequently, we proceeded to characterize further main immune cell subsets before and after conversion from IR-Tac to LCPT (Supplementary Figure S2). Following the switch and the concurrent reduction in tacrolimus, there were no notable changes in the overall percentage of lymphocytes (Supplementary Figure S2A) or in subpopulations like NK cells (CD16^+^CD56^dim^ and CD16^−^CD56^bright^), NKT cells (Supplementary Figures S2C–SE). Granulocyte percentages, encompassing CD16^+^ neutrophils and CD16^−^ eosinophils, similarly showed no significant changes post-switch (Supplementary Figures S1F–SH). Identification of monocyte subtypes based on CD14 and CD16 allowed us to distinguish three categories: classical (CD14^+^CD16^−^), intermediate (CD14^+^CD16^+^), and non-classical monocytes (CD14^dim^CD16^+^). No discernible alterations were observed in these cells between 4 and 8 weeks post-transition from IR-Tac to LCPT (Supplementary Figures S2I–SL). Only the classical monocytes exhibited a significant change after 8 weeks, although this did not translate into a substantial shift in the median values (Supplementary Figures S2H). Finally, the percentage of dendritic cells and their subpopulations did not change after the switch from IR-Tac to LCPT (Supplementary Figure S3).

## Discussion

Switching tacrolimus medication in kidney transplant patients from twice-daily IR tacrolimus to once-daily LCP tacrolimus is associated with a reduction in tacrolimus exposure. Due to the improved bioavailability of LCT-tacrolimus, tacrolimus blood levels remain at the same level as with IR-tacrolimus. In this work, we were able to show that the switch to LCTP and the associated redution of the total amount of tacrolimus by 30% does not lead to significant changes in the composition and activation of the cellular immune system and that the immunosuppressive state is maintained despite the switch from twice-daily to once-daily medication

Several studies have shown that LCP-tacrolimus has a comparable efficacy and safety profile and that switching to LCPT does not lead to increased acute rejection or graft rejection (12, 14, 16, 18, 19). Although the average blood concentration of tacrolimus is the same between IR-tacrolimus and LCP-tacrolimus, there are differences in pharmacokinetics between the two drugs. LCP-tacrolimus exhibits a 30% lower percentage fluctuation in blood concentration from peak to trough, as well as a longer time to reach maximum blood concentration (t_max_) and a lower maximum concentration (C_max_) (20). The extent to which these differences in the pharmakinetics between IR-tacrolimus and LCP-tacrolimus affect the cellular immune system has not been extensively studied.

The immunosuppressive effect of tacrolimus is primarily due to its ability to reduce IL-2 production by inhibiting the transcription factor NFAT through calcineurin inhibition, which occurs downstream of the T-cell receptor. T cells not only express calcineurin and are thus a primary target of tacrolimus, but they are also activated autocrinally by IL-2, making them particularly sensitive to differences in tacrolimus levels (21, 22). We found no differences in the percentage of CD3^+^ T cells or CD4^+^ helper T cells, or CD8^+^ cytotoxic T cells after conversion from IR-tacrolimus to LCP-tacrolimus within the first 4 weeks. In particular, the proliferation of CD4^+^ T helper cells is inhibited by tacrolimus (23). If the switch to LCTP and the associated changes in tacrolimus pharmacokinetics were to lead to a change in immunosuppression, changes would be expected particularly in this T cell population. Our results show, that the immunosuppression of CD4^+^ T cells is stable even after conversion. The pharmacological differences, with a lower tacrolimus C_max_ and t_max_ resulting from a switch to LCP-tacrolimus, have no effect on T cell expansion, which is reflected in the stable frequency of peripheral T cells (24). However, the effect on follicular T helper cells, which express more NFAT (25), appears to be greater than on peripheral CD4^+^ T cells (26) Although follicular T helper cells can be detected in peripheral blood, their frequency is so low that changes are unlikely to be detected when analyzing pan-CD4T cells.

Interestingly, we saw a borderline significant, short-term increase in CD4^+^ regulatory T cells after the switch to LCPT. This was accompanied by a significant shift from memory to naïve phenotype. All of these differences returned to normal after 8 weeks, but show that transient changes in regulatory T cells can occur after the conversion from IR-Tac to LCPT. This study cannot establish a causal relationship between the conversion from IR-Tac to LCPT and the increased percentage of Tregs. It can only be speculated whether the altered tacrolimus formulation led to this effect as LCPT has a significantly longer T_max_ and lower C_max_ concentration compared to IR-Tac and requires only once-daily dosing (13). The resulting consistent tacrolimus level throughout the day without two Tacrolimus-peaks could potentially lead to less inhibition of Tregs. Nevertheless, this must be further investigated, and it must be ruled out that other effects, such as changes in concomitant immunosuppression, have contributed to this effect. The exact effect of tacrolimus on regulatory T cells is not fully understood. While there are studies that show a decrease in regulatory T cells with long-term tacrolimus therapy after pediatric liver and kidney transplantation (27), there are also suggestions that Tregs are less directly affected (28), as the NFAT pathway is less dominant after activation in Tregs compared to CD4^+^ Th cells (29). An increase in Tregs could support immunosuppression and prevent T cell-mediated graft rejection. However, since the changes in Tregs were only of short duration in our study and there were no rejections during the study period, clinical relevance is most likely negligible.

In addition to expansion, the calcineurin-NFAT axis is also crucial for the activation of T cells. After restimulation of T cells with the recall antigen tetanus/diphteria before and after the switch from IR-Tac to LCPT, we did not observe any differences in the expression of the activation markers CD137 and CD154 on T helper cells and CD137 on cytotoxic T cells. Since the transcription factor NF-kB downstream of NFAT is also responsible for the regulation of CD154 (30) and CD137 (31), this shows that switching to LCPT has no effect on the ability and extent of expression of these activation markers by the T cells after stimulation. The transcription factor NF-kB is also responsible for the transcription of a number of effector molecules (22). In addition to IL-2 (32), the binding of the transcription factor to promoters for TNF (33) and interferon-γ (34) has also been demonstrated. However, the expression of these effector molecules after T cell receptor stimulation was not altered after switching to LCPT. This shows that immunosuppression of T cell activity is just as effective after switching from IR-Tac to LCPT and underpins why the overall rate of treatment failures from 11.9% (LCPT) to 13.4% (IR-Tac) with biopsy-proven acute rejections from 8.2% (LCPT) to 9.5% (IR-Tac) was noninferior after switching to LCPT (18).

Intresstingly, 4 weeks after conversion, there was an increase in B cells among the patients. However, the direct effect of the Tacrolimus-molecule is small (6), although B cells express calcineurin and NFat and important genes for development, survival and activation are under the control of NF-kB (35). However, co-cultivation experiments have shown that the inhibitory effect of tacrolimus on B-cells and antibody production is T-cell dependent (36)—probably via direct inhibition of follicular T helper cells (26). It is possible that the pharmacokinetic differences of LCPT led to a temporarily lower inhibition of follicular T helper cells, which would explain the rise in B cells as they are also influenced by tacrolimus therapy. However, we did not see any differences in the differentiation and frequency of memory B cells after switching tacrolimus therapy from IR-Tac to LCPT. And since the frequency of b cells had normalized after 8 weeks and as there were no cases of DSA in the patient group during the study period, no clinical consequences of this short-term B cell expantsion are apparent.

Limitations of the present study include the small patient sample size and the single-center study design. Additionally, due to the exploratory nature of the study, no correction for multiple testing was applied, which may lead to Type I errors. Further studies are needed to investigate and confirm the results and hypotheses generated from this study (37).

In summary, we demonstrated that switching and reducing tacrolimus medication did not result in significant changes to the cellular immune system, nor did it diminish the immunosuppressive effect of tacrolimus. In fact, the transient expansion of regulatory T cells observed could potentially enhance the overall immunosuppressive effect.

## Methods

### Study population and design

Observational trial on 16 renal transplant recipients that are converted from IR-Tac to LCPT for medical reasons (high variability of tacrolimus trough levels, fast metabolizer). Blood samples were drawn before, 4 and 8 weeks after conversion. The analysis involves using flow cytometry to characterize key immune cell populations, various subsets of effector and regulatory T-cells, as well as different stages of B-cell subsets, assessing both their differentiation stage and functional capabilities. Demographics and clinical characteristics of patients are shown in Supplementary Table S1.

### Preparation of PBMCs

Peripheral blood was collected in S-Monovette K3 EDTA blood collection tubes (Sarstedt, Germany). Collected blood was pre-diluted in PBS/BSA (Gibco, Thermo Fisher, USA) at a 1:1 ratio and underlaid with 15 ml Ficoll-Paque Plus (GE Healthcare, USA). Tubes were centrifuged at 800 g for 20 min at room temperature. Isolated PBMCs were washed twice with PBS/BSA and directly stained for flow cytometry analysis or used in functional assay.

### Stimulation with SARS-CoV-2 overlapping peptide pools

Isolated PBMCs were stimulated with 10 µl Tetanus-diphtheria-adsorbate vaccine [TD-pur, Sanofi Pasteur MSD, France. Tetanus-Toxoid ≥40 international unit (IU); diphtheria –Toxoid ≥4 IU per ml] and CD28 (1 µl/ml purified and unlabeled, BD, USA). 2.5 × 10^6^ PBMCs were plated for each condition in 96-UWell Plates in RPMI media (Life Technologies, USA), supplemented with 1% Penicillin-Streptomycin-Glutamine (Sigma Aldrich, USA), and 10% FCS (PAN-Biotech, USA) and were stimulated or left untreated as a control for 16 h. As a positive control, cells were stimulated with SEB (1 µg/ml, Sigma Aldrich, USA) and negative control was with vehicle (a medium to dissolve peptide pools). After 2 h, Brefeldin A (1 µg/ml, Sigma Aldrich, USA) was added. As previously applied by our groups and others, antigen-specific responses were considered positive after the non-specific background was subtracted, and more than 0.001% or at least 15 positive cells were detectable. Negative values were set to zero.

### Antibodies

(all antibodies are from BioLegend unless otherwise noted).

Basic immune status: CD45-A488; clone: 2D1, CD56-PerCP-Cy5.5; clone: NCAM, CD14-PE-Vio770; clone: TÜK4 (Miltenyi Biotec), CD4-A700; clone: OKT4, CD16-APC-Vio770; clone: REA423 (Miltenyi Biotec), CD8-V500; clone: RPA-T8 (BD), CD19-BV605; clone: HIB19, CD45RA-BV650; clone: HI100, CD3-BV785; clone: OKT3, CCR7-PE; clone: G043H7.

B cell Panel: CD138-FITC; clone: MI15, CD27-PE; clone: M-T271, CD21-PE-Cy7; clone: Bu32, IgM-APC; clone: MHM-88, CD45-A700; clone: 2D1, CD14-APC-Vio770; clone: TÜK4 (Miltenyi Biotec), IgD-VioBlue; clone: IgD26 (Miltenyi Biotec), CD20-BV510; clone: 2H7, CD19-BV605; clone: HIB19, CD38-BV650; clone: HB-7, CD3-BV785; clone: OKT3.

Treg Panel: CCR7 (CD197)-AF488; clone: G043H7, CD25-PE-Cy7; clone: 2A3 (BD), CD4-A700; clone: OKT4, LD-eFluor780; vv 1:10; (eBioscience), CD8-V500; clone: RPA-T8 (BD), CD45RA-BV605; clone: HI100, CD127 (IL-7R*α*)-BV650; clone: A019D5, FoxP3-PE; clone: PCH101 (Thermo Fisher Scientific), Helios-A647; clone: 22F6, CD3-BV785; clone: OKT3.

Dendritic cell Panel: CD303 (BDCA-2)-FITC; clone: 201A, CD141 (BDCA-3)-PE; clone: M80, CD123-PE-Dazzle 594; clone: 6H6, CD56-PerCP-Cy5.5; clone: NCAM, CD14-PE-Vio770; clone: TÜK4 (Miltenyi Biotec), CD169 (Siglec-1)-APC; clone: 7-239, CD45-A700; clone: 2D1, LD-eFluor780; vv 1:10; (eBioscience), CD20-APC-Cy7; clone: 2H7, CD1c (BDCA-1)-BV421; clone: L161, CD33-BV510; clone: WM53, CD16-BV605; clone: 3G8, HLA-DR-BV650; clone: L243, CD3-BV785; clone: OKT3.

TD specific T cells: Surface staining: CCR7 (CD197)-PerCP-Cy5.5; clone: G043H7, CD4-A700; clone: OKT4, LD eFluor780 (eBioscience), CD8-V500; clone: RPA-T8 (BD Biosciences), CD45RA-BV605; clone: HI100. Intracellular staining: IL-2-PE; clone: MQ1-17H12, CD137 (4-1BB)-PE-Cy7; clone: 4B4-1, CD154 (CD40l)-A647; clone: 24-31, TNF*α*-eFluor450; clone: MAb11 (eBioscience), IFN*γ*-BV650; clone: 4S.B3, CD3-BV785; clone: OKT3. Fixable Viability Dye eFluor 780 (eBioscience) was used for live/dead discrimination**.**

### Flow cytometry

EDTA-treated whole blood was stained with optimal concentrations of each antibody for 10 min at room temperature in the dark. Erythrocytes were lysed using VersaLyse (Beckman-Coulter) with 2.5% IOTest 3 Fixative Solution (Beckman-Coulter) for 30 min at room temperature in the dark. Panels for Tregs, B cells, DCs and stimulated T cells were extracellular stained with optimal concentrations of antibodies for 10 min at room temperature in the dark. For Treg Panel and stimulated T cells, stained cells were washed twice with PBS/BSA before preparation for intracellular staining using the Intracellular Fixation & Permeabilization Buffer Set (Thermo Fisher Scientific) as per manufacturer's instructions. Fixed and permeabilized cells were stained for 30 min at room temperature in the dark with an optimal dilution of antibodies against the intracellular antigen. All samples were immediately acquired on a CytoFlex flow cytometer (Beckman Coulter). Quality control was performed daily using the recommended CytoFlex Daily QC Fluorospheres (Beckman Coulter). No modification to the compensation matrices was required throughout the study. Flow cytometry data were analyzed using FlowJo version 10.6.2 (BD Biosciences); gating strategies are presented in Supplementary Figures S4–S8.

### Statistical analysis

Statistical analysis was performed using R, version 4.2.1. Categorical variables are summarized as numbers and frequencies; quantitative variables are reported as mean and interquartile range. Violin plots depict the median and the first and third quartiles. All applied statistical tests are paired and two-sided. Differences in quantitative variables between all three groups are analyzed using non-parametric Mann-Whitney *U*-Test. *P* values below 0.050 were considered significant; *P* values were not corrected for multiple testing, as this study was of exploratory nature (37).

## Data Availability

The raw data supporting the conclusions of this article will be made available by the authors, without undue reservation.
