# Supplementary material for: Effects of switching from twice-daily tacrolimus to once-daily extended-release meltdose tacrolimus on cellular immune response
Source: Front Transplant. 2024 Sep 25;3:1405070. doi: 10.3389/frtra.2024.1405070 (PMC11461451; doi:10.3389/frtra.2024.1405070)
Supplement: Supplementary file 1 [file Datasheet1.pdf]

Supplemental Materials

Effects of switching from twice-daily tacrolimus to once-daily extended-release meltdose tacrolimus on cellular immune response

Moritz Anft[1], Panagiota Zgoura[2,3], Sarah Skrzypczyk[1], Michael Dürr[2,3,4,] Richard Viebahn[3], Timm H. Westhoff[1], Ulrik Stervbo[1], Nina Babel[1,4]

[1]Center for Translational Medicine and Immune Diagnostics Laboratory, Medical Department I, Marien Hospital Herne, University Hospital of the Ruhr-University Bochum, Hölkeskampring 40, 44625 Herne, Germany

[2]Clinic for internal medicine, St. Anna Hospital Herne, Hospitalstraße 19, 44649 Herne, Germany

[3]Clinic for surgery, Knappschaftskrankenhaus Bochum, In der Schornau 23-25, 44892 Bochum, Germany

[4]Berlin Institute of Health, Berlin-Brandenburg Center for Regenerative Therapies, and Institute of Medical Immunology, Charité – Universitätsmedizin Berlin, Corporate Member of Freie Universität Berlin, Humboldt-Universität zu Berlin Augustenburger Platz 1, 13353 Berlin, Germany

Supplemental Tables

Supplemental Table S1. Cohort statistics.

	**Kidney Transplant Patients**	
Number (n)	16	
Age	55.4 [41.05-69.2%] *	
Sex (female)	6 [37.5%] ¥	
Transplant age (month)	0.8 [0.4-3%] *	
Transplant type (deceased donor)	14 [87.5%] ¥	
**Immunosuppression**	**Before conversion**	**After conversion**
Tacrolimus	16 [100%] ¥	16 [100%] ¥
Prograf	15 [4 mg] £	0
Crilomus	1 [2 mg] £	0
Envarsus	0	16 [2.25 mg] £
MMF/MMA	14 [1500mg] £	11 [2000mg] £
Glucocorticoide	16 [5 mg] £	16 [5 mg] £
Azathioprin	0	1 [50 mg] £

*median [IQR]; ¥n [%]; £n, [median daily dosis]

Supplemental Figures

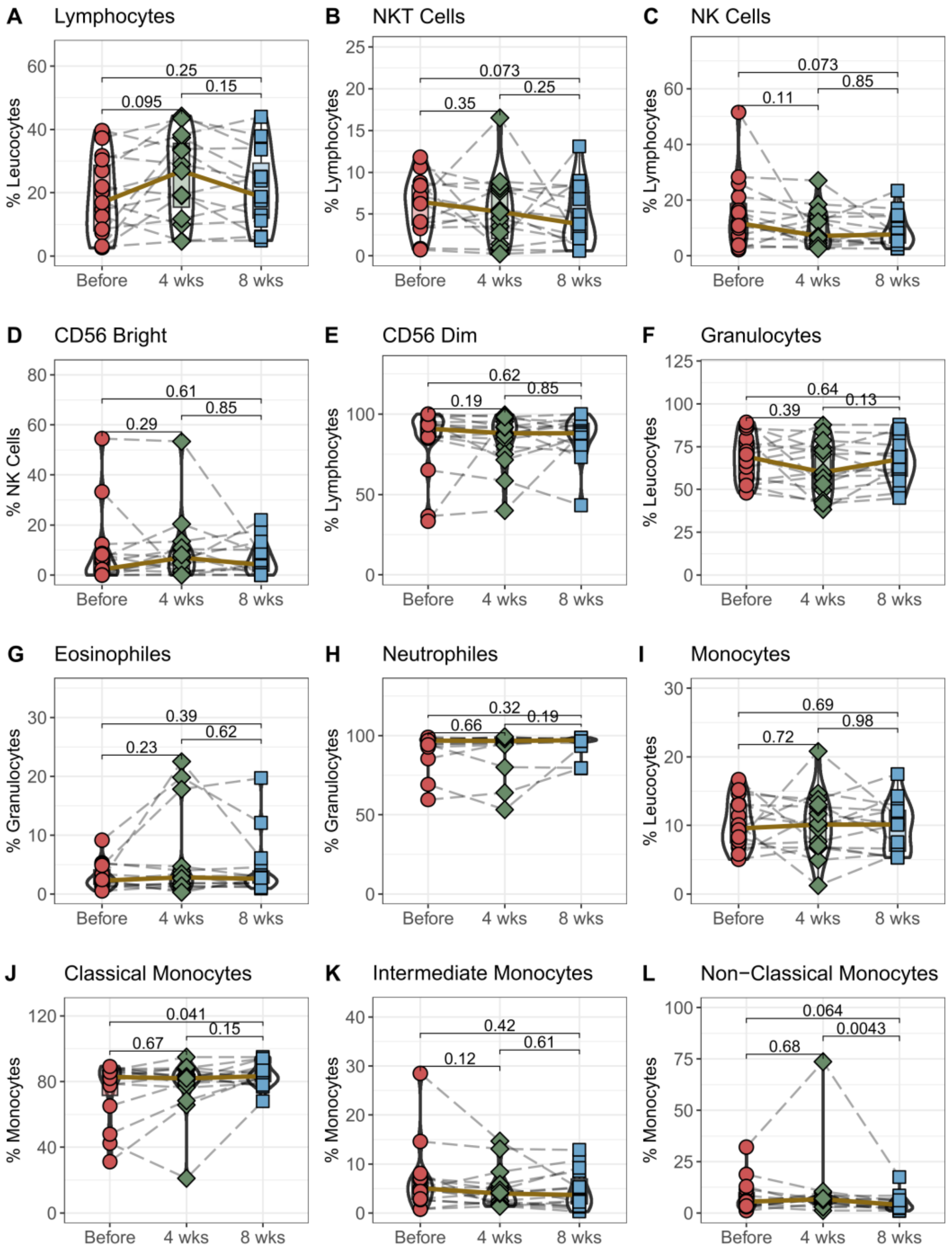

Supplimental Figure S1: Peripheral blood from 16 patients was drawn before IR-Tac -> LCPT conversion, and after 4 and 8 weeks. Whole blood was stained with antibody panel for basic immune cells. Leucocytes were identified by CD45 and Lymphocytes, Monocytes, Granulocytes by characteristic CD45/SSC-profile. B cells were identified as $CD3^{-}CD19^{+}$ Lymphocytes and NKT cells as $CD3^{+}CD56^{+}$ Lymphocytes. NK cells were identified as $CD3^{-}CD56^{+}$ and further distinguished in $CD16^{+}CD56^{Dim}$ NK cells and $CD16^{-}CD56^{Bright}$ NK cells. Monocytes were grouped by expression of CD14 and CD16 in $CD14^{+}CD16^{-}$ classical Monoctyes, $CD14^{+}CD16^{+}$ intermediate Monocytes and $CD14^{dim}CD16^{+}$ non-classical Monocytes. Granulocytes were destinguised in $CD16^{+}$ neutrophiles and $CD16^{-}$ eosinophiles. Gating strategy see Supplemental Figure S3. Mann Whitney U-test (paired) $p<0.05$ = statistically significant.

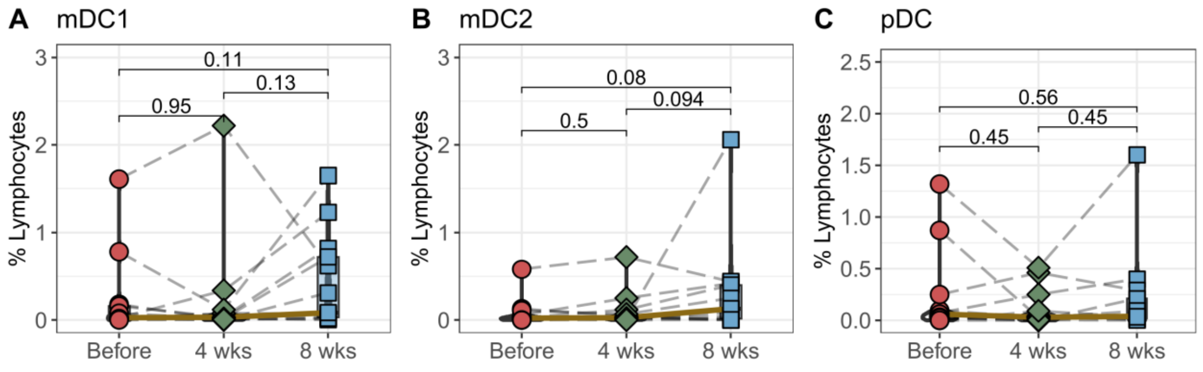

Supplimental Figure S2. Dendritic cells: Peripheral blood from 16 patients was drawn before IR-Tac -> LCPT conversion, and after 4 and 8 weeks. Peripheral blood from patients was drawn before anti CMV-therapy, after 7 and 14 days. Dendritic cells were identified as CD45^{+}SSC^{low}CD20^{-}CD3^{-}CD14^{-}CD56^{-}HLA^{-}DR^{+}. Further, pDCs were identified as CD123^{+} and mDCs as CD123^{low}BDCA3^{+}. MDCs were further distinguished in mDC1 (BDCA1^{-}) and mDC2 (BDCA1^{+}). Gating strategy see Panel and Supplemental Figure S7. Mann-Whitney U Test (paired) $p<0.05$ = statistically significant.

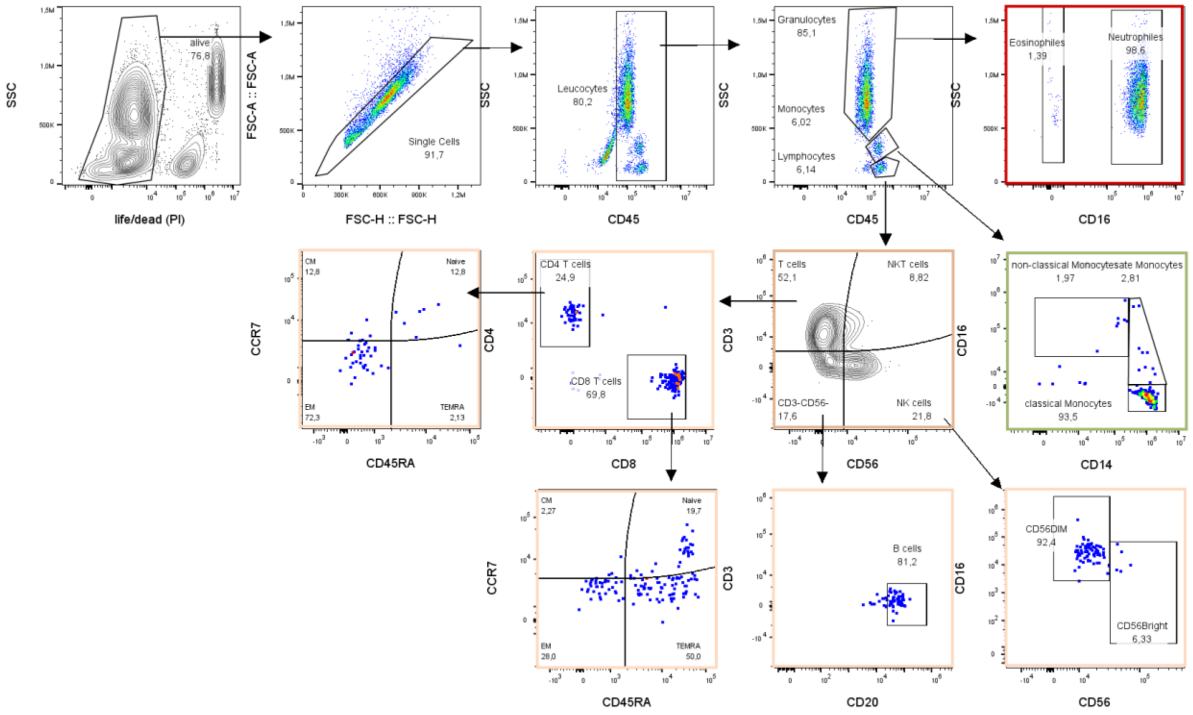

Supplimental Figure S3. Whole blood staining: Analysis of the general immune populations: Granulocytes, monocytes and lymphocytes were distinguished by CD45 and side scatter profile. Granulocytes were separated by CD16 in Eosinophiles ($CD16^{-}$) and Neutrophiles ($CD16^{+}$). Monocytes were divided by the expression of CD14 and CD16 into classical-($CD14^{++}CD16^{-}$), intermediate- ($CD14^{++}CD16^{+}$) and non-classical Monocytes ($CD14^{+}CD16^{+}$). Lymphocytes were separated by CD3 and CD56 in NK ($CD3^{-}CD56^{+}$), NKT ($CD3^{+}CD56^{+}$) and T cells ($CD3^{+}CD56^{-}$) and T cells were further distinguished into $CD4^{+}$ helper T cells and $CD8^{+}$ cytotoxic T cells. Memory T cells were identified as naïve ($CCR7^{+}CD45RA^{+}$), central memory (CM, $CCR7^{+}CD45RA^{-}$) effector memory (EM, $CCR7^{-}CD45RA^{-}$) and T effector memory TA cells (TEMRA, $CCR7^{-}CD45RA^{+}$). B cells were identified as CD3/CD56 double negative and CD19 positive cells.

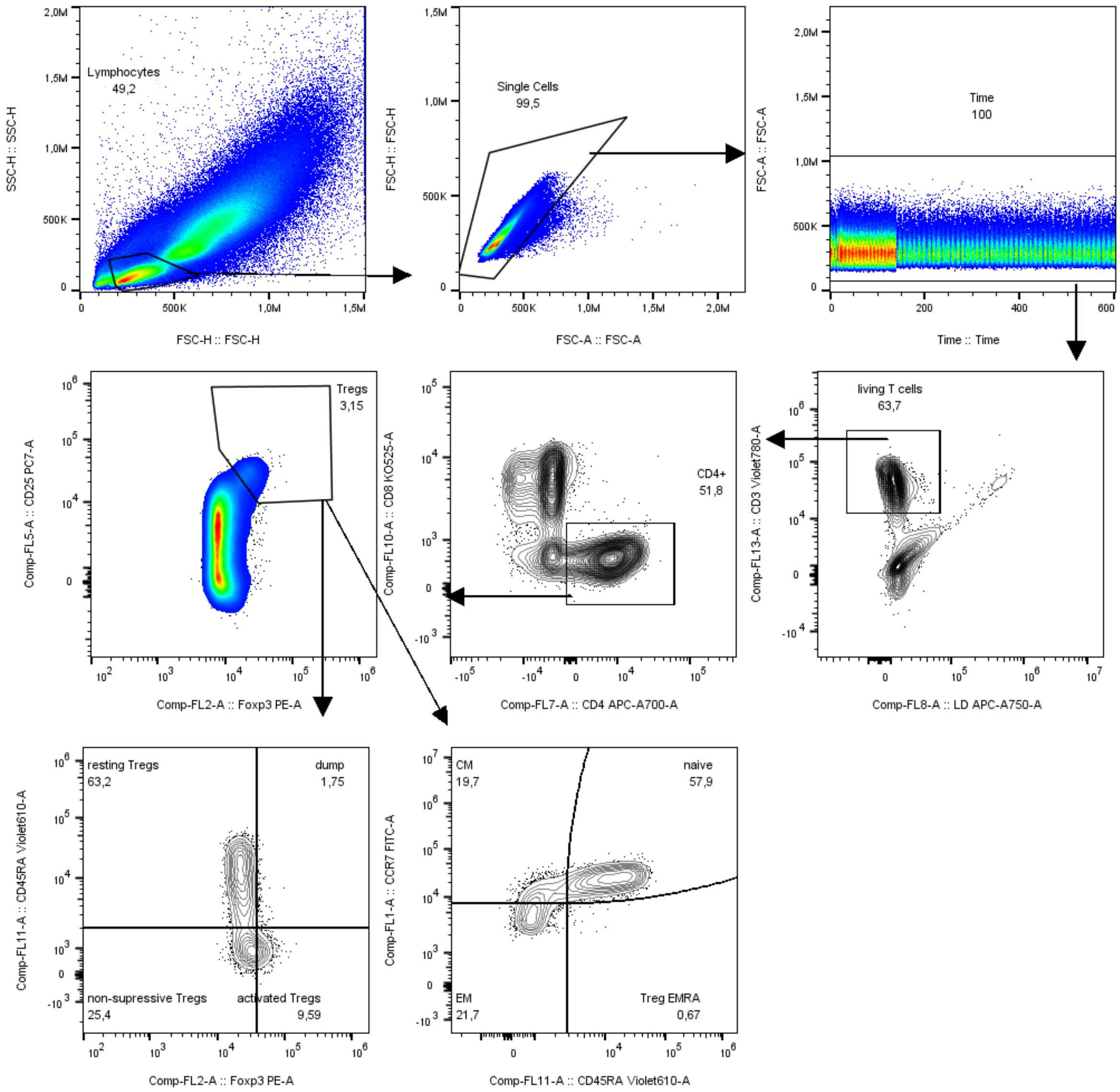

Supplimental Figure S4. Treg Panel: Analyzing of $CD3^+CD4^+CD25^+FoxP3^+$ Tregs. These cells were further analyzed as resting Tregs ($CD45RA^+FoxP3^{low}$), non-supressive Tregs ($CD45RA^-FoxP3^{low}$), and activated Tregs ($CD45RA^-FoxP3^{high}$). Memory Tregs were identified as naïve ($CCR7^+CD45RA^+$), central memory (CM, $CCR7^+CD45RA^-$) effector memory (EM, $CCR7^-CD45RA^-$) and Treg EMRA ($CCR7^-CD45RA^+$).

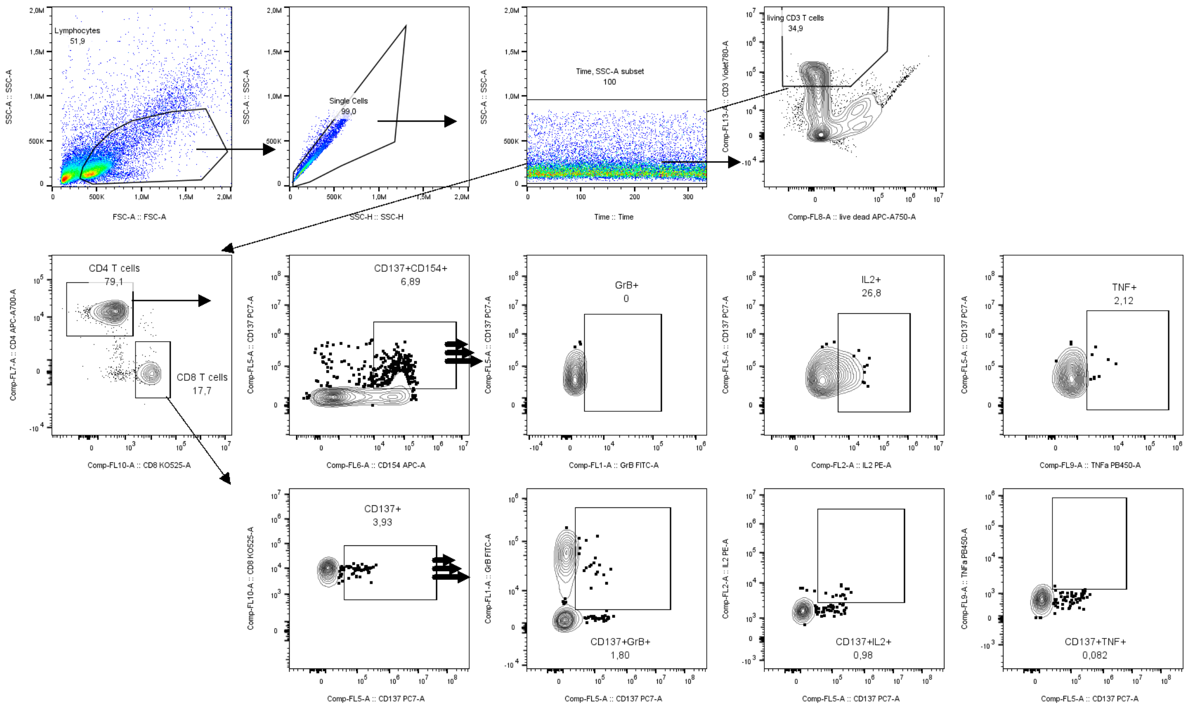

Supplimental Figure S5. T cell stimulation: Isolated PBMCs were stimulated for 16h with TD-vaccin. TD reactive $CD4^{+}CD154^{+}CD137^{+}$ T helper cells and TD reactive $CD8^{+}CD137^{+}$ cytotoxic T cells were identified and further analyzed for the expression of IL-2, TNF and Interferon-γ.

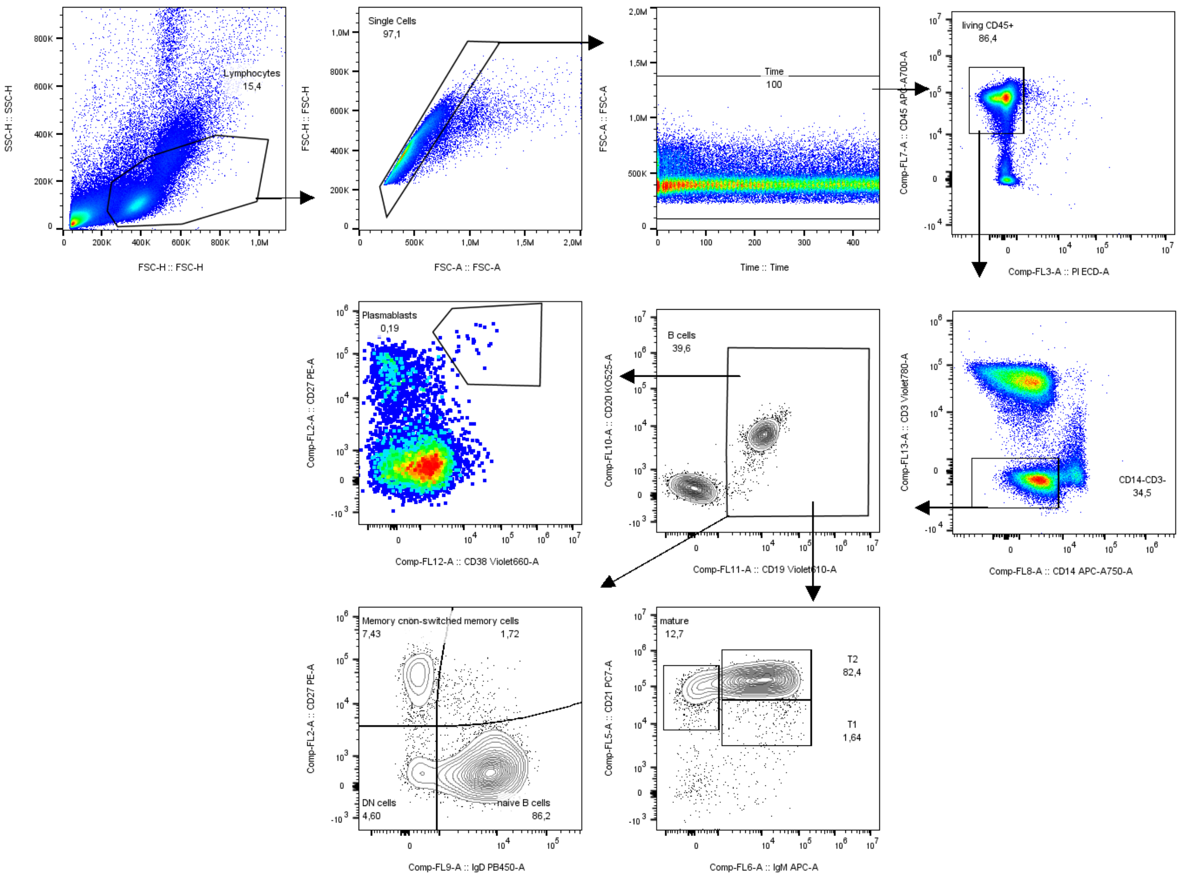

Supplimental Figure S6. B cell Panel: Identification of $CD14^{-}CD3^{-}CD19^{+}CD20^{+}$ B cells and further analyzing of plasmablasts ($CD27^{high}CD38^{high}$). B cell development stages were identified as mature B cells ($IGM^{-}CD21^{+}$), transitional 1 (T1, $IgM^{+}CD21^{-}$) and transitional 2 (T1, $IgM^{+}CD21^{+}$) B cells. B cells were further analyzed for memory subpopulations: Naïve B cells ($IgD^{+}CD27^{-}$), non-switched memory ($IgD^{+}CD27^{+}$), memory B cells ($IgD^{-}CD27^{+}$) and double negative B cells (DN, $IgD^{-}CD27^{-}$).

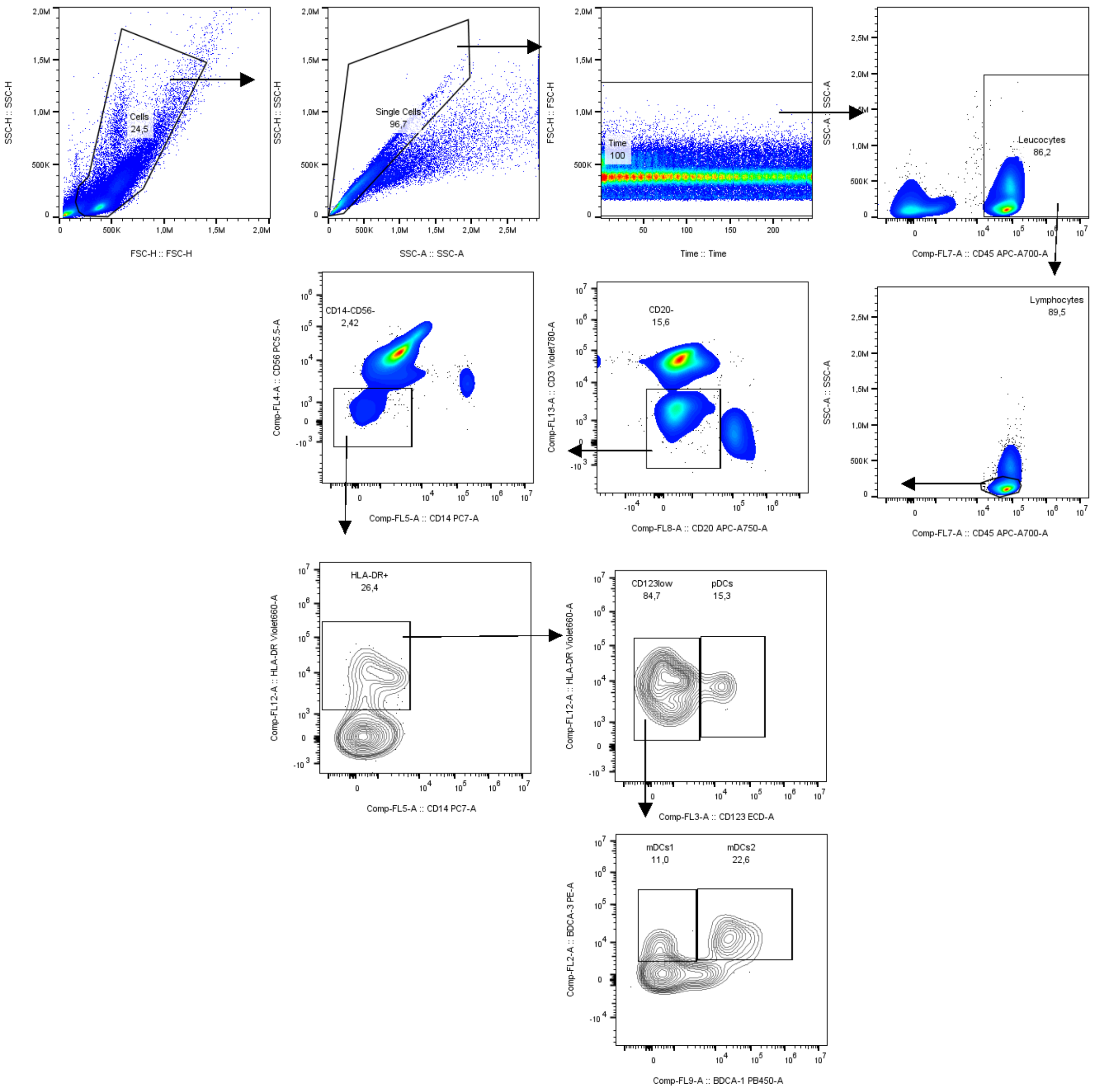

Supplimental Figure S7. Dendritic cell Panel: Dendritic cells were identified as CD45^{+}SSC^{low}CD20^{-}CD3^{-}CD14^{-} CD56^{-}HLA-DR^{+}. Further, pDCs were identified as CD123^{+} and mDCs as CD123^{low}BDCA3^{+}. MDCs were further distinguished in mDC1 (BDCA1^{-}) and mDC2 (BDCA1^{+}).
